# An experimental study of the intrinsic stability of random forest variable importance measures

**DOI:** 10.1186/s12859-016-0900-5

**Published:** 2016-02-03

**Authors:** Huazhen Wang, Fan Yang, Zhiyuan Luo

**Affiliations:** College of Computer Science and Technology, Huaqiao University, Jimei Avenue, Xiamen, 361021 China; Automation Department, Xiamen University, Siming South Road, Xiamen, 361005 China; Computer Learning Research Centre, Royal Holloway, University of London, Egham, Surrey, TW20 0EX UK

**Keywords:** Random forest, Variable importance measure, Stability, Feature selection

## Abstract

**Background:**

The stability of Variable Importance Measures (VIMs) based on random forest has recently received increased attention. Despite the extensive attention on traditional stability of data perturbations or parameter variations, few studies include influences coming from the intrinsic randomness in generating VIMs, i.e. bagging, randomization and permutation. To address these influences, in this paper we introduce a new concept of intrinsic stability of VIMs, which is defined as the self-consistence among feature rankings in repeated runs of VIMs without data perturbations and parameter variations. Two widely used VIMs, i.e., Mean Decrease Accuracy (MDA) and Mean Decrease Gini (MDG) are comprehensively investigated. The motivation of this study is two-fold. First, we empirically verify the prevalence of intrinsic stability of VIMs over many real-world datasets to highlight that the instability of VIMs does not originate exclusively from data perturbations or parameter variations, but also stems from the intrinsic randomness of VIMs. Second, through Spearman and Pearson tests we comprehensively investigate how different factors influence the intrinsic stability.

**Results:**

The experiments are carried out on 19 benchmark datasets with diverse characteristics, including 10 high-dimensional and small-sample gene expression datasets. Experimental results demonstrate the prevalence of intrinsic stability of VIMs. Spearman and Pearson tests on the correlations between intrinsic stability and different factors show that #feature (number of features) and #sample (size of sample) have a coupling effect on the intrinsic stability. The synthetic indictor, #feature/#sample, shows both negative monotonic correlation and negative linear correlation with the intrinsic stability, while OOB accuracy has monotonic correlations with intrinsic stability. This indicates that high-dimensional, small-sample and high complexity datasets may suffer more from intrinsic instability of VIMs. Furthermore, with respect to parameter settings of random forest, a large number of trees is preferred. No significant correlations can be seen between intrinsic stability and other factors. Finally, the magnitude of intrinsic stability is always smaller than that of traditional stability.

**Conclusion:**

First, the prevalence of intrinsic stability of VIMs demonstrates that the instability of VIMs not only comes from data perturbations or parameter variations, but also stems from the intrinsic randomness of VIMs. This finding gives a better understanding of VIM stability, and may help reduce the instability of VIMs. Second, by investigating the potential factors of intrinsic stability, users would be more aware of the risks and hence more careful when using VIMs, especially on high-dimensional, small-sample and high complexity datasets.

## Background

Feature selection is widely used to identify the most discriminating features out of a large number of features in bio-medical applications, such as biomaker discovery, medical diagnosis, and gene selection. Random Forest (RF) is an ensemble classifier, which applies *bagging* technique to construct an ensemble of trees, with *randomization* technique for the growth of each tree [1]. The tree-based ensemble makes RF suitable for handling with both categorical and numerical features, missing values, and redundant features [2]. Especially, RF is suitable for high-dimensional and small-sample datasets [3–6]. RF provides two Variable Importance Measures (VIMs), i.e. the Mean Decrease Accuracy (MDA) and Mean Decrease Gini (MDG). The feature ranking produced by MDA or MDG serves as a filter to eliminate irrelevant features, and has been applied in a large variety of domains [3, 7–11].

It is widely believed that high stability is equally important as high classification accuracy in the feature selection literature [12–18]. The stability of feature selection always refers to the sensitivity of a VIM to data perturbation or parameter variations. With respect to data perturbation stability, the main focus is the consistence between feature rankings, each of which comes from different subsamples of a training set (e.g., 10-fold cross validation) [15, 19–21]. Calle and Urrea discussed the stability of both MDA and MDG rankings based on the variations in a bladder cancer recurrence dataset containing 723 independent features [22]. The average percentage of overlap between the original ranking and the ranking in the perturbed datasets (10 % left out) is used to assess the stability. The conclusion was that MDG is robust to small perturbations of the data while MDA rankings behavior was completely unstable. Nicodemus, K.K kept going deep into the instability of VIMs with respect to data-specific characteristics. Some artificial datasets were generated concerning within-feature relevance and differences in category frequencies [23]. The stability was analyzed by the correlation coefficient between the feature rankings from the original data set and 100 90 % subsamples. The comparison leads to the conclusion that MDG is inferior to MDA on artificial datasets. Verikas et al explored the MDA stability by observing the Spearman coefficient of feature rankings obtained in 20 different runs [24]. Each run performs under the same parameter setting with the training dataset being randomly selected out of the original dataset. Kursa, M.B. compared the stability of four RF-based or RF-relevant VIMs [25]. The stability was assessed among 30 optimal feature subsets derived from 30 bootstrap samples of equal size to the original data. With respect to parameter-variations stability, the studies concentrate on the consistence between feature rankings, when the parameter settings are different from each other [12, 14, 16, 17]. Okun and Priisalu noticed the influence of the number of features for node split on the feature rankings from MDG, where the correlation of two feature rankings was computed, provided before and after the number of features for node split is changed [4]. The results showed the correlation of two feature rankings can be weak while they may exhibit similar accuracy on the same data set. Verikas et al also tried to demonstrate the correlations between a pair of feature rankings generated by a pair of random forests with a very similar number of trees and/or variables (adjacent numbers) [24]. The results showed lower correlations when the number of variables used to split a node in two RFs differs more. In summary, previous studies on the stability of VIMs have tried to attribute the stability problem to the perturbations of training data or parameter settings.

In this paper we address the problem of intrinsic stability which comes from the algorithm design of VIMs. Generally speaking, most feature selection algorithms are relatively stable when eliminating the impacts of data-perturbations or parameter variations, e.g. Support Vector Machine Recursive Feature Elimination [26] and relief-F [27]. However, due to the intrinsic randomness of bagging and randomization, random forest lacks stability decreasing the robustness of performance [28–30]. In our previous work [28], we noticed the intrinsic stability problem of random forest and tried to alleviate it by combining of proximity measure and support vector machine. However, the intrinsic stability problem has not been formally defined and thoroughly investigated, especially the comparison with traditional stability and potential affecting factors. This limitation motivated us to explore the intrinsic stability of VIMs based on random forest. We introduce the concept of *intrinsic stability* which is defined by the self-consistence among the feature rankings of repeated runs. Intrinsic stability describes the stability of VIMs stemming from the intrinsic randomness in algorithm design and distinguishes from traditional stability of data perturbations and parameter variations.

The goal of this study is to explore the intrinsic stability that stems from the intrinsic randomness of VIMs. The experiments were carried out on 19 benchmark datasets with diversified characteristics. Ten of them are gene expression datasets, which are described as high dimensional and small sample problemm, since small sample size and high feature redundancy are important factors that increase randomness [19, 21, 25, 31].

Besides the demonstration of intrinsic stability on a variety of datasets, a more valuable goal of this study is to investigate the influence of several factors on intrinsic stability throughout the VIM process. First, we examined the impact of parameters setting, i.e. the number of trees (ntree) and the number of splitting features candidate for each node (mtry). Second, we investigated the impact of dataset indicators, i.e. the number of features, sample size, the number of classes, and model accuracy. Another highlight of our study is the comparison of magnitude of intrinsic stability with traditional stability, which gives a better understanding of the importance of intrinsic stability.

## Methods

### Random forest variable importance measures

#### Random forest model

Random forest (RF) is an ensemble of multiple decision trees. Each tree of RF is grown with a subset of data made from bootstrap and random subset of variables [1]. The process of sampling a bootstrap data from the original training data to establish the training dataset for each tree is described as bagging technique. The process of selecting a feature subset of the original feature set for tree-node split is described as randomization technique. To classify a new instance, RF puts the new instance down each tree in the forest. Each tree provides a predicted label as a vote for prediction. RF chooses the classification with the most votes. With respect to bagging method, there are on average 36.8 % of original instances not used as the training dataset for each meta tree [1]. All the excluded examples construct the so called out-of-bag dataset (OOB dataset). The OOB accuracy is always applied to evaluate the RF performance. Building on the bagging and randomization technique, RF achieves higher accuracy with low bias and variance than other popular tree structured algorithms like CART, C4.5 and ID3, and has been considered as a highly preferred state-of-art machine learning model [32].

#### MDA and MDG

Considering the learning scenario, the data is described as *z*=(*x*,*y*) where *x* refers to an instance and *y* refers to the label. The instance can further be denoted as *x*=(*x*^1^,*x*^2^,...,*x*^*d*^)∈*X*, with the upper index 1,2,...,*d* representing the original sequence numbers of the features, and *d* is the size (cardinality) of the feature set. When a VIM method is performed, each feature is designated with an importance score. Thus a feature ranking can be obtained by ordering the importance scores. The feature ranking can be described as follows:

(1) $$  RankFea = ({x^{\pi (1)}},{x^{\pi (2)}},...,{x^{\pi (d)}})  $$

where *π*(*j*),*j*=1,2,...,*d* is the new index of feature *x*^*j*^ in the descending ranking.

Building on RF modeling, MDA and MDG have been proposed to serve as variable importance methods. Suppose *h*_*t*_(*x*_*i*_) and ${h_{t}}({x_{i}^{j}})$ refer to the predicted label for OOB instance *x*_*i*_ before and after feature permutation respectively, MDA measures the importance of a feature *x*^*j*^ by calculating the mean decrease in the OOB accuracy before and after the permutation of feature *x*^*j*^, i.e.,

(2) $$  {\fontsize{8.5}{12}{\begin{aligned} VI({x^{j}}) \,=\, {\frac{1} {{n_{tree}}}}\!{\sum\nolimits}_{{\mathrm{t}} = 1}^{{n_{tree}}} {{\frac{\sum\limits_{i \in OOB} {I\left({{y_{i}} = {h_{t}}\left({{x_{i}}} \right)} \right)} - \sum\limits_{i \in OOB} {I\left({{y_{i}} = {h_{t}}\left({{x_{i}^{j}}} \right)} \right)}} {\left| {OOB} \right|}}} \end{aligned}}}   $$

For MDG, we measure the total decrease in node impurities (e.g., Gini index) from splitting on the feature, and average over all trees. Suppose *Gini*(*j*) is the Gini index of feature *x*^*j*^, and *n*_*dot*_ is the number of tree nodes based on feature *x*^*j*^, the importance score by MDG is defined as follows:

(3) $$  VI({x^{j}}) = {1 \over {{n_{dot}}}}\left[ {1 - \sum\limits_{k = 1}^{{n_{dot}}} {Gini{{(j)}^{k}}}} \right]  $$

where *Gini*(*j*)^*k*^ is the *k*^*th*^ Gini index of feature *x*^*j*^ among the *n*_*dot*_ tree nodes.

#### Sources of randomness in MDG and MDA

**Fig. 1:**
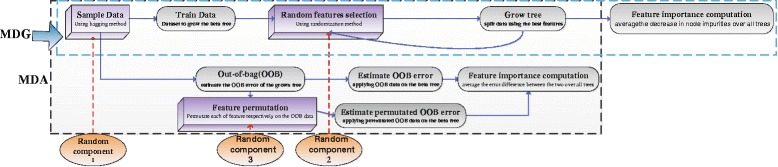
Visualization of random components involved in the procedures of MDG and MDA. The distribution of random components of VIMs is eloquently visualized to understand the source of intrinsic randomness of VIMs

The problem of the reproducibility of RF has received attention [29, 30]. It is pointed out that the stability of RF is reduced by two random components: the bagging method and the randomization method. According to the algorithm mechanism, both MDA and MDG involve the two random components in feature ranking process. Beyond that, one more random component has been involved in MDA, i.e. feature permutation [28]. The random components of VIMs can be eloquently visualized in Fig. 1.

It can be seen in Fig. 1, MDG only includes two random components from RF, i.e., bagging (randomness component 1) and randomization (random component 2). Besides them, MDA involves the third random components of feature permutation (randomness component 3). Knowing the anchor points of random components in VIMs helps understand the sources of intrinsic instability.

### Evaluation criteria for VIM stability

There are a few evaluation criteria aiming to measure the VIM stability [33–36]. Here we propose to measure the consistence between the sequences as a measure of VIM stability. Generally, VIM stability is measured with respect to feature ranking. Three commonly used evaluation criteria, i.e., Spearman coefficient, Jaccard index and Kuncheva index, are applied to comprehensively assess the VIM stability. Among them, Spearman coefficient focuses on the correlation between two sequences, while Jaccard index and Kuncheva index concern the overlap of feature subsets. Moreover, considering the fact that a slight perturbation in feature importance may lead to a dramatic change in feature ranking, mean absolute relative difference (MARD) is also used to evaluate the performance of VIM stability. MARD is often used as a quantitative indicator of quality assurance and quality control for repeated measurements where the outcomes are expected to be the same. The measurement of MARD provides detailed information of VIM stability.

Now consider the general framework for assessing VIM stability among multiple feature rankings. Given *k* feature rankings: *RankFea*^1^,*RankFea*^2^,...,*RankFea*^*k*^, the consistence among the *k* feature rankings is measured by averaging over all pairwise feature rankings, i.e., (*RankFea*^*g*^,*RankFea*^*h*^) where *g*,*h*∈{1,2,...,*k*} and *g*≠*h*. The average consistence is computed as follows:

(4) $$   stabid{x^{k}} \,=\, {{2\!{\sum\nolimits}_{g = 1}^{k - 1} {{\sum\nolimits}_{h = g + 1}^{k} \!{stabid{x^{2}}(RankFe{a^{g}},RankFe{a^{h}})}} } \over {k(k - 1)}}  $$

where *stabidx*^2^(*RankFea*^*g*^,*RankFea*^*h*^) represents an evaluation criterion to measure the pairwise consistence.

It is worth noting that, VIMs are extremely sensitive to redundant or noisy features, especially on high dimensional with a small sample size datasets. It makes sense to only analyze the top ranked features [35, 36]. In this study, we constrain that up to top 100 features submitted to stability evaluation. That means, *stabidx*^2^(*RankFea*^*g*^,*RankFea*^*h*^) is computed with respect to the top 100 features if the length of feature ranking is larger than 100.

#### Spearman coefficient

Spearman coefficient instinctively assesses the rank correlation between two sequences of ranking features [37]. The calculation of Spearman coefficient begins with the process of converting the numerical sequence to ranks. Building on two sorted feature rankings (*RankFea*^*g*^,*RankFea*^*h*^), the Spearman coefficient defined for pairwise consistence can be given by

(5) $$  {stabidx}_{Spearman}^{2} = 1 - 6\sum\limits_{j = 1}^{d} {{{{{\left({RankFe{a_{j}^{g}} - RankFe{a_{j}^{h}}} \right)}^{2}}} \over {d({d^{2}} - 1)}}}  $$

where $RankFe{a_{j}^{g}}$ and $RankFe{a_{j}^{h}}$ are the index of feature *x*^*j*^ in the feature ranking respectively. A preferred value is 1 when the two feature rankings are identical and a value of -1 meaning that they have exactly inverse orders. According to the limit of up to top 100 features, *d* is set to be 100 if the length of feature ranking is larger than 100.

#### Jaccard index

Jaccard index is widely used in the literature of stability evaluation, which calculates the similarity between pairs of feature rankings concerning the aspect of overlap [38]. For two sorted feature rankings (*RankFea*^*g*^,*RankFea*^*h*^), Jaccard index is defined as the size of the intersection of two sequences divided by the size of the union of the two sequences. The Jaccard index definitely will be 1 when the numerator and denominator are both 1. Therefore, in order to correct this problem, an alternate Jaccard index, which iterates through each sub-sequence and then averages the aggregated results from all steps, is given as follows:

(6) $$   {stabidx}_{Jaccard}^{2} = {1 \over {d - 1}}\sum\limits_{j = 1}^{d - 1} {{{\left| {{RankFea}_{{1}...j}^{g} \cap {RankFea}_{{1}...j}^{h}} \right|} \over {\left| {{RankFea}_{{1}...j}^{g} \cup {RankFea}_{{1}...j}^{h}} \right|}}}  $$

where ${RankFea}_{{1}...j}^{g},{RankFea}_{{1}...j}^{h}$ are the sub-sequence of the original feature rankings (*RankFea*^*g*^,*RankFea*^*h*^). Jaccard index takes value in [0,1]. The closer that number is to 1, the better the VIM stability is. According to the limit of up to top 100 features, *d* is changed to be 100 if the length of feature ranking is larger than 100.

#### Kuncheva index

Kuncheva index is a more sensitive measure than Jaccard index, which can correct the evaluation bias [33]. It is pointed out that Jaccard index tends to produce higher values for larger subsets due to the increased bias of selecting overlapping features by chance. Kuncheva index tends to provide a correction for chance. For two sorted feature rankings (*RankFea*^*g*^,*RankFea*^*h*^), the computation iterates through each sub-sequence and then averaged evaluation is defined as

(7) $$  {stabidx}_{Kuncheva}^{2} = {\frac{1}{d-1}}\sum\limits_{j = 1}^{d - 1} \frac{{r_{j}} - \left({j^{2} / d} \right)}{j - \left(j^{2} d \right)}  $$

where *r*_*j*_ is the cardinality of intersection of sub-sequences ${RankFea}_{{1}...j}^{g}$ and ${RankFea}_{{1}...j}^{h}$. Kuncheva index takes a value in [−1,1]. Larger value indicates larger number of common features in both sub-sequences. According to the limit of up to top 100 features, *d* is set to be 100 if the length of feature ranking is larger than 100.

#### MARD

The evaluation criterion of mean absolute relative difference (MARD) is a frequently used measure of the differences between two sequences of real values [39]. Basically, the MARD represents the standard deviation of the differences between two sequences. MARD is a good measure of consistence of two sequences with respect to real values. For two sequences of importance score *IMS*^*g*^ and *IMS*^*h*^, MARD calculates the difference of absolute values of importance score between sequences as follows:

(8) $$  {stabidx}_{MARD}^{2} = {1 \over d}\sum\limits_{j = 1}^{d} \frac{\left| {{s_{j}^{g}} - {s_{j}^{h}}} \right|}{({s_{j}^{g}} + {s_{j}^{h}})/2}  $$

where ${s_{j}^{g}}, {s_{j}^{h}}$ represent the elements of scores sequence *IMS*^*g*^,*IMS*^*h*^, respectively.

It is worth noting that the calculation of MARD up to the top 100 features is somewhat complicated. In this study, The sequence of importance score is obtained by the union of pairwise sequences with up to top 100 features. Therefore, there may be more than 100 features involved in each sequence of importance scores. Accordingly, *d* is the united length of the two sequences when the original feature ranking is larger than 100.

## Datasets and experimental setup

In order to provide a more convincing empirical verification, various types of datasets were chosen. Most of the datasets in this study are collected from the biology domain and have the characteristics of small sample-size and high-dimensional features. Table 1 shows a summary of the 19 data sets used. Among them, 14 of the total 19 datasets comes from the application of biology, and 11 from gene expression datasets except *Arcene* and *madelon*, are obtained from a repository of the most widely studied gene expression sets (http://www.gems-system.org/) [40]. The dataset Arcene, madelon and the rest are obtained from UCI Machine Learning Repository (http://archive.ics.uci.edu/ml/).

**Table 1:** Characteristics of datasets used in experiments

ID	Dataset	Domain	#Feature	#Sample	#Class	OOB accuracy
1	yeast	biology	8	1484	10	0.98
2	glass	Physical	9	240	6	0.79
3	vote	social	16	232	2	0.97
4	segment	image	19	2310	7	0.98
5	mushroom	biology	20	8124	2	1.00
6	soybean	biology	35	307	19	0.93
7	splice	biology	60	3175	4	0.43
8	sonar	Physical	60	208	2	0.85
9	Madelon	artificial	500	2600	2	0.73
10	SRBCT	biology	2308	83	4	1.00
11	Leukemia1	biology	5327	72	3	0.94
12	DLBCL	biology	5469	77	2	0.83
13	Tumors_9	biology	5726	60	9	0.51
14	Brain_Tumor1	biology	5920	90	5	0.83
15	Arcene	biology	10000	100	2	0.79
16	Brain_Tumor2	biology	10367	50	4	0.74
17	Prostate_Tumor	biology	10509	102	2	0.92
18	Tumors_11	biology	12533	174	11	0.88
19	Lung_Cancer	biology	12600	203	5	0.92

Four dataset indicators are used to describe the characteristics of datasets. Besides three commonly used statistics, i.e., #feature, #sample and #class, the fourth indicator OOB accuracy is used to evaluate the complexity of a dataset [41]. The OOB accuracy of each dataset is the best result of RF on the original dataset with fine-tuned parameters. The implementation of RF model, as well as the runs of MDA and MDG, is executed in the R environment (http://cran.r-project.org/) by calling for the R package of *randomForest4.6-10* [42].

In our experiments, the intrinsic stability is assessed by the self-consistence of the results in repeated 10 runs. The self-consistence among the 10 feature rankings are evaluated respectively by Spearman coefficient, Jaccard index, and Kuncheva index, while the difference of 10 sequences of importance scores is measured by MARD. The illustration of intrinsic stability was conducted in three stages. First, in order to get a stable performance of VIMs, the impact of parameter setting was explored. Second, the correlations between four dataset indicators and the intrinsic stability are statistically investigated. Finally, the magnitude of intrinsic stability was compared with that of the traditional stability with respect to data perturbations and parameter variations.

## Results

### Influence of the parameter setting on intrinsic stability

**Fig. 2:**
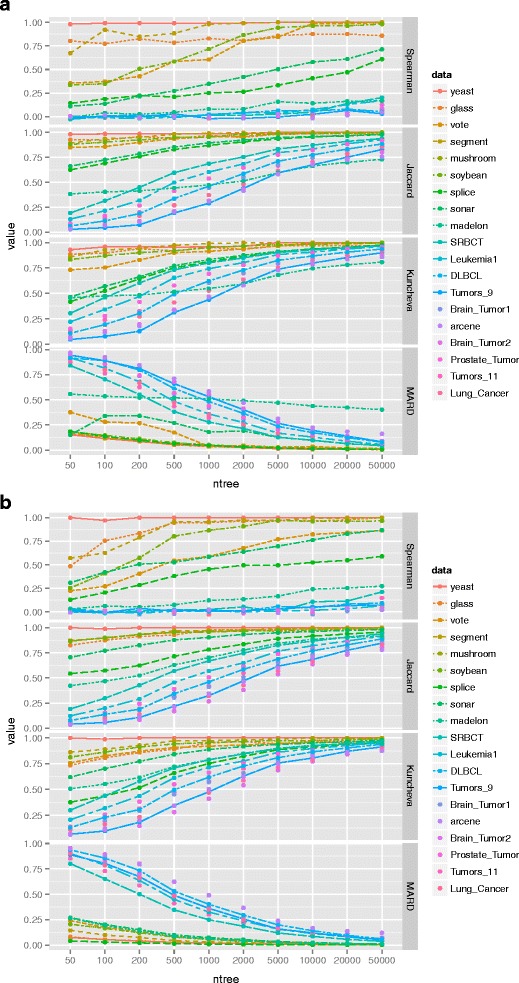
Influence of the setting of parameter ntree on the intrinsic stability. For each dataset, the distribution of intrinsic stability against different values of ntree are illustrated (**a**) MDA (**b**) MDG

**Fig. 3:**
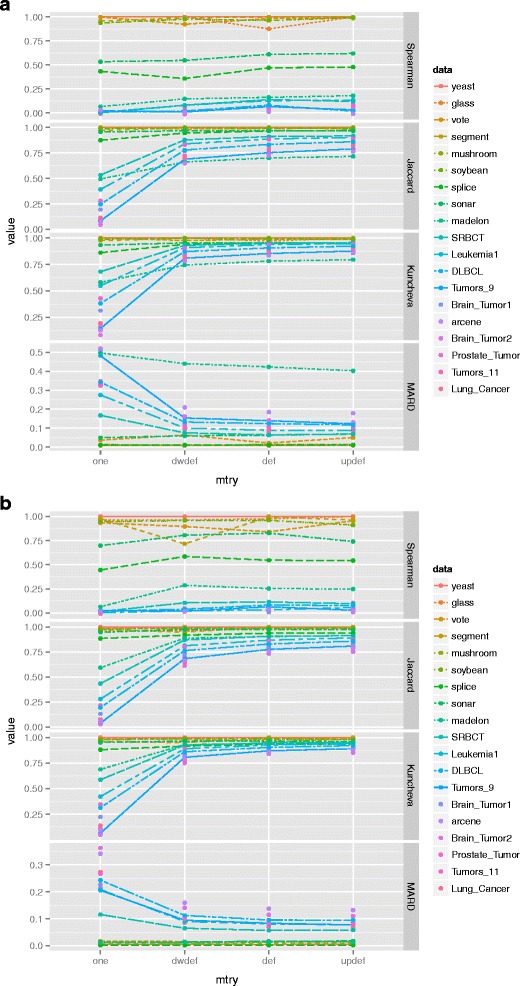
Influence of the setting of parameter mtry on the intrinsic stability. For each dataset, the distribution of intrinsic stability against different values of mtry are illustrated (**a**) MDA (**b**) MDG

In order to explore whether or not the intrinsic stability is affected by the parameter setting of VIMs, the distribution of intrinsic stability against different parameter settings are investigated. The two key parameters ntree and mtry are set to different values respectively. The range of ntree is set as (50, 100, 200, 500, 1000, 2000, 5000, 10000, 20000 and 50000) and the range of mtry is set as (one,*dwdef*,*def*, *updef*), where def means the default value of mtry i.e. the square-root of the total number of features, dwdef means a half of def and updef means one and a half of def. For each dataset, the distribution of intrinsic stability against different values of ntree with the value of mtry being *def* is displayed in Fig. 2. The distribution of intrinsic stability against different values of mtry with ntree being 20000 is displayed Fig. 3. It is worth noting, there are two set of stability indices presented in our study. The first group includes Spearman coefficient, Jaccard index and Kuncheva index, which are based on feature ranking and prefer to be as high as 1. The other is MARD, which is based on the scores of feature importance and prefers to be as low as zero.

It can be seen from Fig. 2, for both MDA and MDG, the intrinsic stability is significantly more obvious to parameter ntree. With the increase of ntree, Spearman coefficient, Jaccard index and Kuncheva index ascend gradually and MARD gradually declines. It shows that the role of ntree nonlinearly decreases with its increasing value. Note that, even when ntree equal to 50000, the values of indices on most of the datasets are still away from the preferred value, which is 1 for the stability indices based on feature rankings and 0 for MARD, especially for datasets with small-size examples and high-dimensional features.

In contrast, Fig. 3 shows that the parameter mtry has little impact on the performance of intrinsic stability. It remained stable against different values of mtry across the 19 datasets. Similar to the results of ntree, the magnitudes of intrinsic stability are always away from the preferred value. Especially, the intrinsic stability on datasets with small-size examples and high-dimensional features tend to be poorer than others.

### The demonstration of intrinsic stability on different datasets

The intrinsic stability across all the 19 datasets are investigated under predefined parameter settings. According to the finding in Figs. 2 and 3, to eliminate the influence of parameters we set ntree to be 20000 and mtry to be the default settings of *def*. The results are respectively shown in Table 2 for MDA and Table 3 for MDG. In each table the performance of stability index is described as its mean and variance over all possible 45 pairwise computations.

**Table 2:** The performance of intrinsic stability of MDA

	Spearman coefficient		Jaccard index		Kuncheva index		MARD	
	Mean	Variance	Mean	Variance	Mean	Variance	Mean	Variance
Yeast	1.0000	0.0000	1.0000	0.0000	1.0000	0.0000	0.0076	0.0000
Glass	0.8756	0.0181	0.9852	0.0003	0.9708	0.0010	0.0077	0.0000
Vote	0.9851	0.0004	0.9909	0.0001	0.9866	0.0002	0.0270	0.0003
Segment	1.0000	0.0000	1.0000	0.0000	1.0000	0.0000	0.0110	0.0000
Mushroom	0.9628	0.0025	0.9925	0.0001	0.9910	0.0001	0.0134	0.0000
Soybean	0.9628	0.0025	0.9925	0.0001	0.9910	0.0001	0.0134	0.0000
Splice	0.4712	0.0162	0.9651	0.0000	0.9548	0.0001	0.0107	0.0000
Sonar	0.6107	0.0170	0.9672	0.0000	0.9509	0.0001	0.2936	2.8892
Madelon	0.1675	0.0104	0.7306	0.0002	0.8072	0.0002	0.4387	2.1041
SRBCT	0.1397	0.0078	0.9103	0.0001	0.9496	0.0000	0.0683	0.0000
Leukemia1	0.1282	0.0087	0.8864	0.0001	0.9370	0.0000	0.0963	0.0001
DLBCL	0.0809	0.0067	0.8333	0.0001	0.9059	0.0000	0.1402	0.0001
Tumors_9	0.0665	0.0084	0.7519	0.0003	0.8528	0.0001	0.1608	0.0002
Brain_Tumor1	0.0176	0.0094	0.8182	0.0002	0.8956	0.0001	0.1148	0.0001
Arcene	0.0453	0.0085	0.7563	0.0003	0.8574	0.0001	0.2283	0.0003
Brain_Tumor2	0.0427	0.0084	0.7378	0.0002	0.8361	0.0001	0.1637	0.0001
Prostate_Tumor	0.0437	0.0115	0.8826	0.0001	0.9362	0.0000	0.1170	0.0001
Tumors_11	0.0134	0.0111	0.8168	0.0002	0.8945	0.0001	0.0808	0.0000
Lung_Cancer	0.0220	0.0146	0.7839	0.0002	0.8745	0.0001	0.0906	0.0001

**Table 3:** The performance of intrinsic stability of MDG

	Spearman coefficient		Jaccard index		Kuncheva index		MARD	
	Mean	Variance	Mean	Variance	Mean	Variance	Mean	Variance
yeast	1.0000	0.0000	1.0000	0.0000	1.0000	0.0000	0.0042	0.0000
Glass	0.9763	0.0010	0.9887	0.0002	0.9778	0.0009	0.0040	0.0000
Vote	0.8408	0.0222	0.9927	0.0001	0.9587	0.0014	0.0161	0.0000
Segment	0.9944	0.0001	0.9974	0.0000	0.9975	0.0000	0.0083	0.0000
Mushroom	0.9601	0.0014	0.9852	0.0002	0.9857	0.0002	0.0141	0.0000
Soybean	0.9601	0.0014	0.9852	0.0002	0.9857	0.0002	0.0141	0.0000
Splice	0.5471	0.0073	0.9380	0.0001	0.9355	0.0001	0.0022	0.0000
Sonar	0.8273	0.0030	0.9733	0.0000	0.9643	0.0001	0.0172	0.0000
Madelon	0.2731	0.0082	0.9158	0.0001	0.9481	0.0000	0.0145	0.0000
SRBCT	0.1154	0.0103	0.9067	0.0001	0.9469	0.0001	0.0596	0.0000
Leukemia1	0.0329	0.0086	0.8684	0.0002	0.9258	0.0001	0.0864	0.0001
DLBCL	0.0832	0.0089	0.8295	0.0001	0.9025	0.0000	0.1036	0.0000
Tumors_9	0.0655	0.0128	0.7753	0.0002	0.8694	0.0001	0.0894	0.0001
Brain_Tumor1	0.0085	0.0107	0.8003	0.0001	0.8828	0.0001	0.0970	0.0000
Arcene	0.0342	0.0115	0.7803	0.0002	0.8729	0.0001	0.1549	0.0001
Brain_Tumor2	0.0297	0.0120	0.7328	0.0003	0.8396	0.0001	0.1262	0.0001
Prostate_Tumor	0.0796	0.0120	0.8580	0.0002	0.9211	0.0001	0.0985	0.0001
Tumors_11	0.0761	0.0143	0.8052	0.0003	0.8877	0.0001	0.0744	0.0000
Lung_Cance	0.0421	0.0101	0.7538	0.0003	0.8547	0.0002	0.0905	0.0000

It can be seen in Table 2 with respect to Spearman coefficient, Jaccard index and Kuncheva index, most of values in terms of the mean are smaller than 1, and the scores in terms of MARD do not touch the bottom of zero. These observations illustrate the prevalence of inconsistence among the results in repeated runs. Especially, the values on gene expression datasets are significantly more obvious than other datasets, which reveals that VIMs on datasets with small-size samples and high dimensional features are more likely to suffer from intrinsic instability. Additionally, all the values in terms of variance are as small as zero, which indicates that the results from different pairwise computations are consistent. According to Table 3, the performance of MDG is analogous to that of MDA.

### Correlation between the dataset indicators and intrinsic stability

In this section, we analyze the correlation between the indicators of dataset characteristics and the intrinsic stability with the purpose of better understanding of the potential factors that may affect the intrinsic stability. The indicators including the number of features, sample size, OOB accuracy and number of classes are studied respectively. In our experiments, two correlation coefficients, i.e., Spearman coefficient and Pearson coefficient, are both used to capture the relationship. Spearman benchmarks monotonic relationship while Pearson coefficient benchmarks linear relationship. For each correlation test, the performance is described as estimate and p value, which is tested with confidence of 95 %.

A preliminary test on the dependencies between different indicators on the 19 datasets showed that #feature and #sample is not independent. (Spearman correlation coefficient for #feature and #sample is -0.63, with a p-value of 0.0038). Specifically speaking the datasets in Table 1 can be divided into two categories of datasets: a) low-dimensional with a large number of samples which is the former 9 datasets in Table 1. b) high-dimensional with a small sample size the latter 10 datasets in Table 1. To eliminate the interference we study the role of feature and sample independently on these two groups respectively. The results in terms of Spearman coefficient and Pearson coefficient are displayed in Table 4 for datasets(a) and Table 5 for datasets(b) respectively. Further more, to investigate the coupling effect of #feature and #sample on the whole 19 datasets, we evaluate the relationship between intrinsic stability and a synthetic indicator #feature/ #sample, which can be seen as an indicator of degree of high dimensional and small sample of the dataset. Tables 6 and 7 show the relationships between intrinsic stability and #feature/ #sample as well as #class and OOB accuracy for MDA and MDG respectively.

**Table 4:** The correlation between datasets indicators and intrinsic stability in datasets(a)

Coefficient	Dataset indicators	Spearman coefficient		Jaccard index		Kuncheva index		MARD	
		estimate	p.value	estimate	p.value	estimate	p.value	estimate	p.value
Spearman	#feature(MDA)	-0.7848	0.0122	-0.7004	0.0356	-0.7004	0.0356	0.6891	0.0401
	#feature(MDG)	-0.8571	0.0031	-0.9244	0.0004	-0.6387	0.0641	0.3445	0.3639
	#sample(MDA)	-0.1345	0.7302	0.0168	0.9658	0.1345	0.7302	-0.1590	0.6828
	#sample(MDG)	-0.0921	0.8138	-0.2594	0.5003	-0.0084	0.9830	-0.4435	0.2318
Pearson	#feature(MDA)	-0.8346	0.0051	-0.9972	0.0000	-0.9755	0.0000	0.8511	0.0036
	#feature(MDG)	-0.8677	0.0024	-0.8289	0.0058	-0.4856	0.1851	0.2730	0.4772
	#sample(MDA)	-0.0319	0.9350	-0.0606	0.8769	-0.0157	0.9681	-0.0966	0.8047
	#sample(MDG)	-0.0769	0.8441	-0.1563	0.6880	0.0520	0.8942	0.0058	0.9883

**Table 5:** The correlation between datasets indicators and intrinsic stability in datasets(b)

Coefficient	Dataset indicators	Spearman coefficient		Jaccard index		Kuncheva index		MARD	
		estimate	p.value	estimate	p.value	estimate	p.value	estimate	p.value
Spearman	#feature(MDA)	-0.8424	0.0045	-0.5030	0.1434	-0.5030	0.1434	0.0424	0.9186
	#feature(MDG)	-0.1879	0.6076	-0.5758	0.0878	-0.5758	0.0878	0.2606	0.4697
	#sample(MDA)	-0.5152	0.1328	0.1636	0.6567	0.1636	0.6567	-0.4424	0.2042
	#sample(MDG)	0.2121	0.5599	0.0667	0.8648	0.0667	0.8648	-0.1152	0.7588
Pearson	#feature(MDA)	-0.7873	0.0069	-0.4687	0.1718	-0.4535	0.1880	0.1424	0.6946
	#feature(MDG)	-0.2785	0.4359	-0.6094	0.0615	-0.5942	0.0701	0.3507	0.3205
	#sample(MDA)	-0.5141	0.1284	-0.0242	0.9471	0.0126	0.9725	-0.4052	0.2453
	#sample(MDG)	0.0359	0.9216	-0.1836	0.6117	-0.1733	0.6321	-0.2310	0.5207

**Table 6:** The correlation between the dataset indicators and intrinsic stability on whole datasets for MDA

Coefficient	Dataset indicators	Spearman coefficient		Jaccard index		Kuncheva index		MARD	
		estimate	p.value	estimate	p.value	estimate	p.value	estimate	p.value
Spearman	#feature/#sample	-0.8227	0.0000	-0.7717	0.0001	-0.7805	0.0001	0.7196	0.0005
	#classes	0.0162	0.9474	0.1868	0.4438	0.1976	0.4173	-0.5079	0.0264
	OOB accuracy	0.4289	0.0669	0.6639	0.0019	0.6498	0.0026	-0.4701	0.0423
Pearson	#feature/#sample	-0.7187	0.0005	-0.7212	0.0005	-0.6408	0.0031	0.2260	0.3522
	#classes	0.2913	0.2263	0.2038	0.4028	0.2428	0.3166	-0.3893	0.0995
	OOB accuracy	0.3246	0.1751	0.3903	0.0985	0.4815	0.0368	-0.2814	0.2432

**Table 7:** The correlation between the dataset indicators and intrinsic stability on whole datasets for MDG

Coefficient	Dataset indicators	Spearman coefficient		Jaccard index		Kuncheva index		MARD	
		estimate	p.value	estimate	p.value	estimate	p.value	estimate	p.value
Spearman	#feature/#sample	-0.8583	0.0000	-0.8530	0.0000	-0.8425	0.0000	0.8969	0.0000
	#classes	0.1524	0.5333	0.0649	0.7917	0.0902	0.7134	-0.3175	0.1853
	OOB accuracy	0.4006	0.0892	0.4930	0.0320	0.5387	0.0173	-0.2230	0.3589
Pearson	#feature/#sample	-0.7503	0.0002	-0.8641	0.0000	-0.8426	0.0000	0.8790	0.0000
	#classes	0.2843	0.2381	0.1416	0.5630	0.1918	0.4315	-0.2371	0.3283
	OOB accuracy	0.2622	0.2782	0.3188	0.1833	0.3989	0.0907	-0.1025	0.6762

Table 4 shows the results for datasets(a). For #feature, the performance are same regardless of MDA or MDG. That is, the estimates of Spearman coefficient and Pearson coefficient are all negative in terms of the stability indices based on feature ranking and positive based on MARD. Meanwhile, most of their p values are all below the significance level 5 %. This observation reflects that the number of features basically performs both negative monotonic correlation and negative linear correlation with the intrinsic stability. When it comes to #sample in terms of both Spearman coefficient and Pearson coefficient, the p values are all higher than the significance level 5 %. From Table 5 which shows the results for datasets(b) with respect to both #feature and #sample, most of the p values are significantly higher than the significance level 5 %, except that the #feature in case of Spearman coefficient for MDA shows both negative monotonic correlation and negative linear correlation. This implies a complicated and ambiguous relationship between intrinsic stability and #feature as well as #sample for high dimensional and small sample datasets.

As shown in Table 6 with respect to the synthetic indictor #feature/#sample, the estimates of Spearman coefficient and Pearson coefficient are all negative in terms of the stability indices based on feature ranking and positive based on MARD, with their p values all below the significance level 5 %. This observation reflects that the synthetic indictor #feature/#sample performs both negative monotonic correlation and negative linear correlation with the intrinsic stability. This implies that high dimensional and small sample datasets are prone to intrinsic instability of VIMs. When it comes to #class all the p values are higher than the significance level 5 %, which indicates that there is no significant correlation between the number of classes and the intrinsic stability. The results of OOB accuracy in the case of Spearman coefficient are not consistent. The p value of the stability index in terms of Spearman coefficient is over 5 % while that of Jaccard index, Kuncheva index and MARD are below the significance level of 5 %. In the case of Pearson coefficient, only Kuncheva index has p value below the significance level. The performance in terms of OOB accuracy leads us to conclude that there is only monotonic correlation between OOB accuracy and intrinsic stability. This implies that data complexity may have impact on the intrinsic stability of VIMs. From Table 7, we find similar performance except for the results of OOB accuracy. It shows only the p values in terms of Spearman coefficient between OOB accaracy, Jaccard index and Kuncheva index are below 5 %, which reveals a weak monotonic correlation between the MDG intrinsic stability and the OOB accuracy. Remembering the importance scores of MDG which is not calculated by OOB accuracy but by Gini index, the mechanism of importance score calculation contributes to this observation.

### Comparison of intrinsic stability and data-perturbation stability

**Fig. 4:**
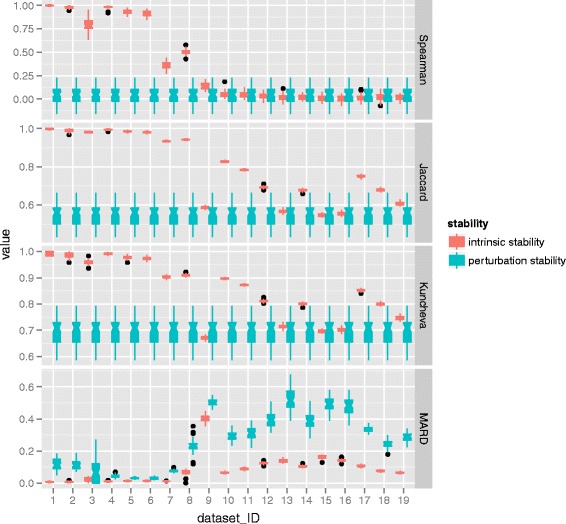
Comparison of intrinsic stability and data-perturbation stability with respect to MDA. For each dataset, a comparison of the distributions of two kinds of stability is presented, one comes from intrinsic stability and the other refers to data-perturbation stability. The distribution is depicted by the notched box which focuses on the variation in the distribution

**Fig. 5:**
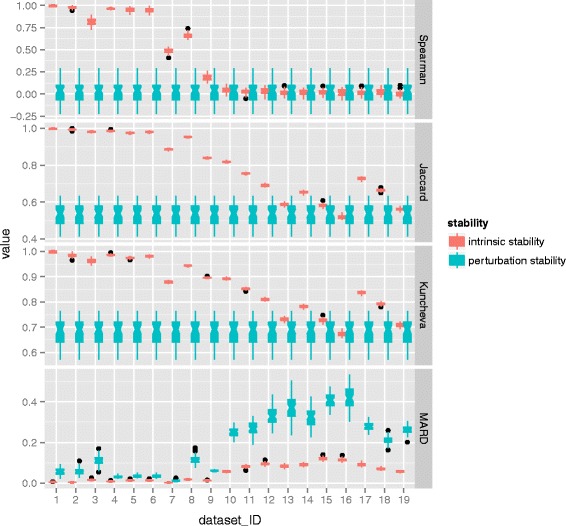
Comparison of intrinsic stability and data-perturbation stability with respect to MDG. For each dataset, a comparison of the distributions of two kinds of stability is presented, one comes from intrinsic stability and the other refers to data-perturbation stability. The distribution is depicted by the notched box which focuses on the variation in the distribution

In this section, the comparison of intrinsic stability and data-perturbation stability are conducted. The data perturbation is conducted by 10-fold cross validation. To do this, an original dataset is randomly partitioned into 10 equal sized data subsets, 9 of the 10 data subsets are used as training set to produce a feature ranking. This process is repeated 10 times, each of which includes different folds as the training dataset. The 10 lists of feature importance scores are then used to compute Spearman coefficient, Jaccard index, Kuncheva index and MARD. Then the average over the 45 pairwise computations are recorded. For intrinsic stability 10 runs of VIMs are executed on each training set, and the stability indices on that training set are computed. Finally, the averaged results over all the 10 training sets is reported. The comparison of intrinsic stability and data-perturbation stability of MDA are displayed in Fig. 4 and the results of MDG are displayed in Fig. 5.

The results are depicted with notched box plot. Each notched box plot displays the variation in the distribution of data based on some statistical summaries; the central rectangle spanning the first quartile to the third quartile (the interquartile range or IQR), the lines extending vertically from the hinge to the highest value (upper whiskers) is within 1.5 times of IQR, the lower whisker extends from the hinge to the lowest value within 1.5 times of IQR. Data beyond the end of the whiskers are outliers and are plotted as individual points. Additionally, the notch is a segment around the median displaying the a confidence interval, with a height of 3.14 times the height of the central box divided by the square root of the number of data elements in the corresponding data distribution. The notch is useful for determining whether two distributions are drawn from the same population. Similar notches of boxes indicate that the data visualized by the boxes have the same distribution. Besides, if the notches of two boxes do not overlap this is strong evidence that the medians differ.

As shown in the case of both MDA and MDG, the positions of boxes referring to intrinsic stability are always higher than that of perturbation stability in terms of Spearman coefficient, Kuncheva index and Jaccard index, while the situation is reversed in terms of MARD. However, the notches of boxes referring to intrinsic stability overlap that of perturbation stability in some cases. For example, the overlap appears in terms of Kuncheva index for MDA, and the situation happens in terms of Spearman coefficient. Additionally, some notches go outside the hinges, such as the notches in terms MARD for both MDA and MDG, the notches in terms of Spearman coefficient for MDG. This is because the size of the notch is bigger than the interquartile range. In other words, the distributions of intrinsic stability or perturbation stability are not symmetric but skewed. This finding reveals that intrinsic stability or perturbation stability are not always normally distributed. Especially, the difference between the intrinsic stability and data-perturbation stability on mushroom dataset are substantially small. Considering the unavoidable intrinsic stability, the observation on mushroom dataset reveals that the major component of data-perturbation stability of mushroom is intrinsic stability. The tendency of splice dataset is similar to that of mushroom dataset. Comparatively, the gaps with respect to mushroom dataset are substantially smaller than that of splice dataset. The most obvious reason for the observation is the good characteristic of mushroom, which has large sample size and high OOB accuracy. For the comparison of MDA and MDG, the size of the box with respect to MDG in terms of MARD is substantially larger than that of MDA. This observation reveals that there exists high variability in the distributions of MDG.

### Comparison of intrinsic stability and parameter-variation stability

In this section the magnitude of intrinsic stability is compared with that of parameter-variations stability. Considering two parameters ntree and mtry are required for VIMs, the comparison is conducted from two aspects.

**Fig. 6:**
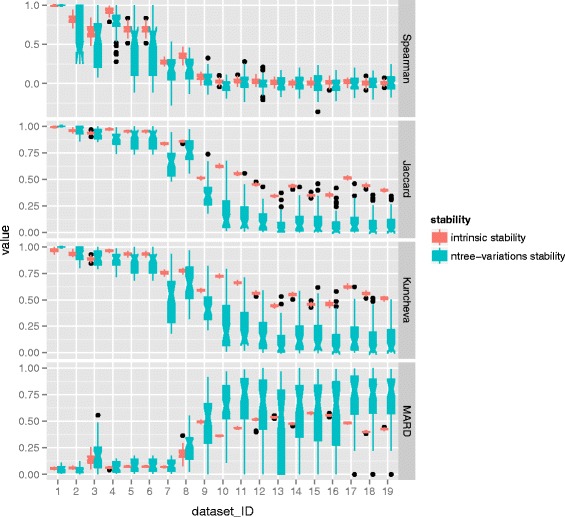
Comparison of intrinsic stability and ntree-variations stability with respect to MDA. For each dataset, a comparison of the distributions of two kinds of stability is presented, one comes from intrinsic stability and the other refers to ntree-variations stability. The distribution is depicted by the notched box which focuses on the variation in the distribution

**Fig. 7:**
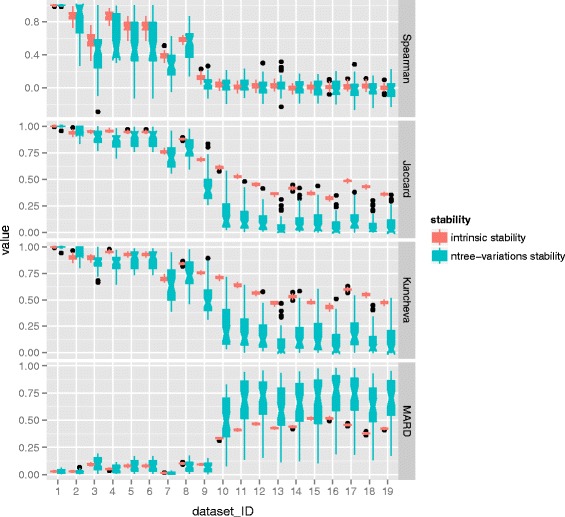
Comparison of intrinsic stability and ntree-variations stability with respect to MDG. For each dataset, a comparison of the distributions of two kinds of stability is presented one comes from intrinsic stability and the other refers to ntree-variations stability. The distribution is depicted by the notched box which focuses on the variation in the distribution

First, the comparison is carried out between the intrinsic stability and the ntree-variations stability. To do so the parameter ntree takes 10 different values with the range of (50, 100, 200, 500, 1000, 2000, 5000, 10000, 20000 and 50000). In this scenario, the parameter mtry is set default *def*. Based on each ntree setting, the VIM can be conducted. The 10 lists of feature importance scores are then used to compute ntree-variation stability. For intrinsic stability, 10 repeated runs are executed under each setting of ntree. The results of stability evaluation based on 10 different settings of ntree are collected and then are averaged. The performance of stability is presented by the distributions of all possible 45 points by pairwise computations. The distributions are then depicted by notched box plot. For each dataset, the comparison of intrinsic stability and ntree-variations stability was conducted. The results of all 19 datasets were illustrated. The results of MDA are displayed in Fig. 6 and the results of MDG can be found in Fig. 7.

It can be seen from Fig. 6 and Fig. 7, generally speaking the positions of boxes referring to intrinsic stability are always higher than that of parameter stability in terms of Spearman coefficient, Kuncheva index and Jaccard index, while the situation is reversed in terms of MARD. Meanwhile, the notches of intrinsic stability do no overlap that of ntree-variations stability. Additionally, some notches go outside the hinges which reveals that the distribution of data is not symmetric but skewed. But beyond that, a remarkable characteristic is that the sizes of box with respect to ntree-variations stability are substantially larger than that of intrinsic stability. This observation reveals that there exists high variability in the distributions of ntree-variations stability.

**Fig. 8:**
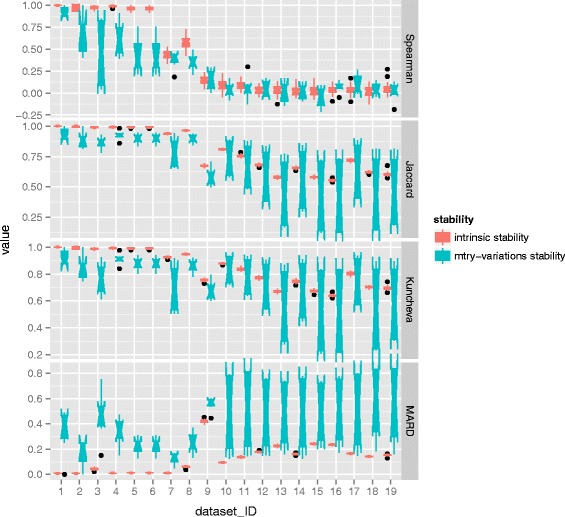
Comparison of intrinsic stability and mtry-variations stability with respect to MDA. For each dataset, a comparison of the distributions of two kinds of stability is presented, one comes from intrinsic stability and the other refers to mtry-variations stability. The distribution is depicted by the notched box which focuses on the variation in the distribution

**Fig. 9:**
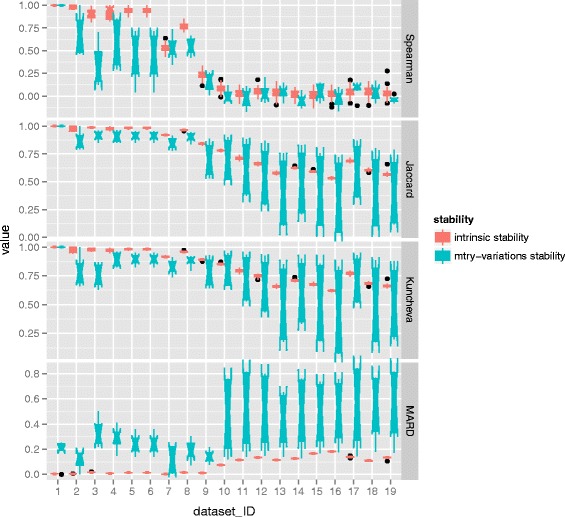
Comparison of intrinsic stability and mtry-variations stability with respect to MDG. For each dataset, a comparison of the distributions of two kinds of stability is presented, one comes from intrinsic stability and the other refers to mtry-variations stability. The distribution is depicted by the notched box which focuses on the variation in the distribution

Second, similar comparison is carried out between the intrinsic stability and mtry-variations stability. To do so the parameter mtry changes its values: one, *dwdef*, *def* and *updef*. The value of def means the square-root of the total number of features, dwdef means a half of def, and updef means one and a half of def. In this scenario, the value of ntree is set as default 20000. Similar computations are conducted to get the results. The results of MDA are displayed in Fig. 8 and the results of MDG can be found in Fig. 9.

It can be seen from Figs. 8 and 9, generally speaking there is no any clear tendency with respect to the positions and the overlap between boxes referring to intrinsic stability and that of mtry-variations stability. Meanwhile, the notches of intrinsic stability do no overlap that of mtry-variations stability. Additionally, the comparison in terms of Spearman coefficient in case of both MDA and MDG, as well as the comparison in term of MARD in case of MDA is obscure, which shows that the positions of boxes referring to intrinsic stability are almost as high as that of parameter stability. Moreover, the notches of intrinsic stability and that of mtry-variations stability are mutually overlapping.

## Discussion

Experimental results show that intrinsic instability is prevalent across different datasets. Particularly, the degree of intrinsic stability is dramatically low in the case of gene expression datasets. The influence of parameter setting of VIMs on the intrinsic stability is investigated and the observations and conclusions are presented as follows:

- With the increase of ntree, the intrinsic stability gets better. Nevertheless, even when ntree equal to 50000, the values of indices on most of the datasets are still away from the preferred value 1. These observations lead to the conclusion that intrinsic instability is inevitable, but can be reduced by a larger value of ntree.
- There is no clear tendency of the distribution of intrinsic stability against different settings of mtry. This observation indicates that the setting of mtry is not a solution to control the intrinsic instability.

With respect to four data-specific indicators, i.e., the number of features, the sample size, the number of classes and OOB accuracy, our observations and conclusions are summarized as follows:

- The relationships between #sample and intrinsic stability can not be observed. For the #feature, there is a perfect monotone decreasing relationship, as well as strong negative linear correlation with intrinsic stability on low-dimensional datasets with a large number of samples. However, only #feature in case of Spearman coefficient for MDA shows both negative monotonic correlation and negative linear correlation on high dimensional datasets with small sample size. This implies a complicated and ambiguous relationship between intrinsic stability and #feature for high dimensional and small sample datasets.
- Tests on the whole 19 datasets show that #feature and #sample have a coupling effect on the intrinsic stability. The synthetic indictor #feature/#sample performs both negative monotonic correlation and negative linear correlation with the intrinsic stability. This implies that high dimensional and small sample datasets are prone to intrinsic instability of VIMs. This effect may stem from the intrinsic randomness in the mechanism of VIMs, the feature randomization (random component 2 in Fig. 1) for both MDA and MDG, as well as the feature permutation (random component 3 in Fig. 1) for MDA.
- Generally, the OOB accuracy have a clear monotonic correlation with the intrinsic stability. However, there is no linear correlation. This observation reveals that data complexity does have impacts on the intrinsic stability.
- There is no significant correlation between the number of classes and the intrinsic stability.

Further, the magnitude of intrinsic stability is compared with that coming from data perturbation or parameter variations. The observations and conclusions are summarized as follows:

- The magnitude of intrinsic instability is generally smaller than that of data-perturbation instability. This observation indicates that data-perturbation stability may contain intrinsic stability.
- The magnitude of intrinsic instability is significantly smaller than that of ntree-variations instability. Moreover, the intrinsic stability has a dramatically smaller variability than that of ntree-variability stability. It shows that the intrinsic stability may be involved in the ntree-variations stability and VIMs is more sensitive to the change of ntree.
- The magnitude of intrinsic stability is generally smaller than that of mtry-variations stability. Nevertheless, there still exists the observation that intrinsic stability and mtry-variations stability have nearly equal magnitude in terms of Spearman coefficient. Besides both intrinsic stability and mtry-variations stability have significantly low variability. These observations reveal that the intrinsic stability is involved in the mtry-variations stability, but mtry has little impact on the stability of VIMs.

Additionally, comparison of MDA and MDG exhibits a lot of similarities between them. They both suffer from the issue of intrinsic stability. Comparatively, MDG performs relatively high variability in terms of MARD while always making a consistent conclusion with the stability indices based on feature ranking. The difference between MDA and MDG lies in the degree of intrinsic stability. Nevertheless, from an overall perspective, there is not any clear conclusion about which one is more stable. The observation is consistent with previous studies. In the research of Calle and Urrea, MDG is more robust than MDA to small perturbations of the data [22]. However, Nicodemus, K.K concluded that MDG is inferior to MDA on artificial datasets [23]. According to the mechanism of intrinsic randomness, the number of random components in Fig. 1 cannot completely depict the behavior of MDA and MDG. Seemingly, MDG involves less random components than MDA. Whereas, Fig. 1 only focuses the breath of random components and does not consider the intensity of each component. It is better to consider the quantity and intensity of random components to evaluate the intrinsic stability of VIMs on different implementations.

## Conclusion

In this paper, a new concept of intrinsic stability of variable importance measures (VIMs) is introduced to concern the influence of intrinsic randomness in algorithm design. The intrinsic stability in VIMs based on random forest MDA and MDG, are comprehensively investigated which assesses the self-consistence between the feature rankings of repeated runs. First, the prevalence of intrinsic stability of VIMs over many real-world datasets demonstrates that the instability of VIMs not only comes from data perturbations or parameter variations, but also stems from the intrinsic randomness of VIMs. The fact that the magnitude of intrinsic stability is always smaller than that of traditional stability indicates that the intrinsic stability is implicitly involved in traditional stability. This finding gives a better understanding of VIM stability, and may help reduce or eliminate the instability of VIMs. Studies towards stable and robust VIMs without regard to the intrinsic randomness of VIMs may not be likely to make any real progress. Second, by investigating the potential affecting factors of intrinsic stability, users would be more aware of the risks and hence more careful when using VIMs, especially on high-dimensional, small-sample and high complexity datasets. In practice a large enough value of ntree is preferred.

## References

[1] Breiman L (2001). Random forests. Mach Learn.

[2] Reif DM, Motsinger AA, McKinney BA, Crowe JE, Moore JH (2006). Feature selection using a random forests classifier for the integrated analysis of multiple data types. Computational Intelligence and Bioinformatics and Computational Biology, 2006. CIBCB’06. 2006 IEEE Symposium On.

[3] Díaz-Uriarte R, De Andres SA (2006). Gene selection and classification of microarray data using random forest. BMC Bioinformatics.

[4] Okun O, Priisalu H (2007). Random forest for gene expression based cancer classification: overlooked issues. Pattern Recognition and Image Analysis.

[5] Statnikov A, Wang L, Aliferis CF (2008). A comprehensive comparison of random forests and support vector machines for microarray-based cancer classification. BMC Bioinformatics.

[6] Boulesteix AL, Janitza S, Kruppa J, König IR (2012). Overview of random forest methodology and practical guidance with emphasis on computational biology and bioinformatics. Wiley Interdiscip Rev: Data Min Knowl Discov.

[7] Lee SS, Sun L, Kustra R, Bull SB (2008). Em-random forest and new measures of variable importance for multi-locus quantitative trait linkage analysis. Bioinformatics.

[8] Altmann A, Toloşi L, Sander O, Lengauer T (2010). Permutation importance: a corrected feature importance measure. Bioinformatics.

[9] Ma D, Xiao J, Li Y, Diao Y, Guo Y, Li M (2011). Feature importance analysis in guide strand identification of micrornas. Comput Biol Chem.

[10] Cao DS, Liang YZ, Xu QS, Zhang LX, Hu QN, Li HD (2011). Feature importance sampling-based adaptive random forest as a useful tool to screen underlying lead compounds. J Chemometrics.

[11] Paul J, Verleysen M, Dupont P (2013). Identification of statistically significant features from random forests. ECML Workshop on Solving Complex Machine Learning Problems with Ensemble Methods.

[12] Yu L, Ding C, Loscalzo S (2008). Stable feature selection via dense feature groups. Proceedings of the 14th ACM SIGKDD International Conference on Knowledge Discovery and Data Mining.

[13] Loscalzo S, Yu L, Ding C (2009). Consensus group stable feature selection. Proceedings of the 15th ACM SIGKDD International Conference on Knowledge Discovery and Data Mining.

[14] He Z, Yu W (2010). Stable feature selection for biomarker discovery. Comput Biol Chem.

[15] Yu L, Han Y, Berens ME (2012). Stable gene selection from microarray data via sample weighting. IEEE/ACM Trans Comput Biol Bioinformatics (TCBB).

[16] Han Y, Yu L (2012). A variance reduction framework for stable feature selection. Stat Anal Data Min: The ASA Data Science Journal.

[17] Kamkar I, Gupta SK, Phung D, Venkatesh S (2014). Stable feature selection for clinical prediction: Exploiting icd tree structure using tree-lasso. Journal of biomedical informatics.

[18] Park CH, Kim SB (2015). Sequential random k-nearest neighbor feature selection for high-dimensional data. Expert Syst Appl.

[19] Kalousis A, Prados J, Hilario M (2007). Stability of feature selection algorithms: a study on high-dimensional spaces. Knowl Inform Syst.

[20] Haury AC, Gestraud P, Vert JP (2011). The influence of feature selection methods on accuracy, stability and interpretability of molecular signatures. PloS one.

[21] Kim SY (2009). Effects of sample size on robustness and prediction accuracy of a prognostic gene signature. BMC Bioinformatics.

[22] Calle ML, Urrea V (2011). Letter to the editor: Stability of random forest importance measures. Brief Bioinformatics.

[23] Nicodemus KK (2011). Letter to the editor: On the stability and ranking of predictors from random forest variable importance measures. Briefings in bioinformatics.

[24] Verikas A, Gelzinis A, Bacauskiene M (2011). Mining data with random forests: A survey and results of new tests. Pattern Recognit.

[25] Kursa MB (2014). Robustness of random forest-based gene selection methods. BMC Bioinformatics.

[26] Guyon I, Weston J, Barnhill S, Vapnik V (2002). Gene selection for cancer classification using support vector machines. Mach Learn.

[27] Zhang Y, Ding C, Li T (2008). Gene selection algorithm by combining relieff and mrmr. BMC Genomics.

[28] Wang H, Wang C, Lv B, Pan X (2015). Improved variable importance measure of random forest via combining of proximity measure and support vector machine for stable feature selection. J Inform Comput Sci..

[29] Boulesteix AL, Bender A, Bermejo JL, Strobl C. Brief Bioinform. 2012; 13(3):292–304. 10.1093/bib/bbr053 21908865

[30] Genuer R (2012). Variance reduction in purely random forests. J Nonparametric Stat.

[31] Cadenas JM, Garrido MC, MartíNez R (2013). Feature subset selection filter–wrapper based on low quality data. Expert Syst Appl.

[32] Kulkarni VY, Sinha PK (2013). Random forest classifiers: a survey and future research directions. Int J Adv Comput.

[33] Kuncheva LI (2007). A stability index for feature selection. Artificial Intelligence and Applications.

[34] Alelyani S, Zhao Z, Liu H (2011). A dilemma in assessing stability of feature selection algorithms. High Performance Computing and Communications (HPCC), 2011 IEEE 13th International Conference On.

[35] Fagin R, Kumar R, Sivakumar D (2003). Comparing top k lists. SIAM J Discrete Math.

[36] Boulesteix AL, Slawski M (2009). Stability and aggregation of ranked gene lists. Brief Bioinformatics.

[37] Fieller EC, Hartley HO, Pearson ES (1957). Tests for rank correlation coefficients. i.. Biometrika.

[38] Hamers L, Hemeryck Y, Herweyers G, Janssen M, Keters H, Rousseau R (1989). Similarity measures in scientometric research: the jaccard index versus salton’s cosine formula. Inform Process Manag.

[39] Pleus S, Schmid C, Link M, Zschornack E, Klötzer HM, Haug C (2013). Performance evaluation of a continuous glucose monitoring system under conditions similar to daily life. J Diabetes Sci Technol.

[40] Statnikov A, Tsamardinos I, Dosbayev Y, Aliferis CF (2005). Gems: a system for automated cancer diagnosis and biomarker discovery from microarray gene expression data. Int J Med Inform.

[41] Ho TK (2002). A data complexity analysis of comparative advantages of decision forest constructors. Pattern Anal Appl.

[42] Liaw A, Wiener M. The randomForest package. Software manual. 2003. https://cran.r-project.org/web/packages/randomForest/.

